# The New Ogilvie Children's Home

**Published:** 1913-05-10

**Authors:** 


### THE NEW OGILVIE CHILDREN'S HOME.

### The Co-operation between Health and Education.

The establishment of the new Ogilvie Children's Home or School for Recovery at Clacton-on-Sea, which was opened in April last, marks an epoch in the history of the co-operation between health and education interests in this country. It is an innovation which deserves the active sympathy of all educational authorities, and which is well worthy of imitation elsewhere. For not only is it the first well-appointed Home of Recovery to which is also attached a School which has been established in England, but it is an institution founded on such broad and generally acceptable principles that it should appeal to everyone who has at heart the physical and intellectual welfare of the rising generation.

The Home was erected and equipped by the Trustees of the Friends' organisation known as "Mrs. Ogilvie's Charities" from designs drawn up by Mr. Rowntree. It provides accommodation for forty children, twenty of each sex, and for the education of these children while they are inmates of the establishment. It is, therefore, a School as well as a Home, and it is the intention of the Trustees to cater for as wide a field as possible within the limitations of their space and accommodation capacity. In the general statement which has been forwarded to educational authorities in the counties of Essex, Suffolk, and London, for the children of which counties the School is primarily intended, the objects and scope of the institution are clearly outlined. It is stated that "the institution is intended to be the means of setting up into a condition of permanent good health some of the many delicate children who frequent the elementary schools and whose ailments are neither so apparent nor so serious as to demand immediate hospital treatment, yet who are on so low a physical level that they would otherwise be prevented from eventually being able to meet the demands of industrial life effectively. Such cases, in fact, as the anæmic, rheumatic, ill-nourished children so often found in the public elementary schools, who, if neglected, are liable to contract heart disease, pulmonary tuberculosis, hip or spine disease, or one of the other complaints associated with the neglect of a delicate physique in early life. . . ." This object, it is safe to say, is a very laudable and desirable one. Indeed, the wonder is that the public has not before now grasped the importance of providing some institution which is available for the treatment, on common-sense lines, of these borderland cases.

It is a well recognised fact that a school child sent for a prolonged period to a seaside or convalescent home benefits in health at the expense of his or her education. Perhaps it is true to say that the child's health is the primary consideration; but it is well to bear in mind in these days of competition and industrial stress that the neglect of school education materially affects the after-life of the child. The ideal home is one of this kind in which, while the health interests of the children are looked after, their education is also regarded as an important consideration, and adequate provision is made for suitable teaching. That being the case, the Ogilvie School at Clacton will be cordially welcomed, especially as it appears to be under most excellent management and control. The School is in charge of a matron, who is a properly trained nurse and who is responsible to the Trustees. An additional resident nurse will be provided, and the School will have the services of a specially appointed medical man, who will attend periodically.

These provisions show that every care has been taken to provide for the physical supervision of the children, especially when it is remembered that the School is not intended for children who are suffering from ailments which would necessitate any great amount of individual attention or nursing or who are crippled or disabled. It will cater for the feeble, ill-nourished child, of the type styled by school doctors as "pretubercular," and it is just in regard to these children who, while suffering from no well-defined lesion, are yet in want of good nourishment, fresh air, and a healthy, hygienic environment, that such homes are desirable. The age limit of children admitted will be from eight to twelve years, and only in exceptional circumstances will a child be retained in the institution after he has passed the age of twelve years. This, again, is a necessary and desirable limitation. "No child will be accepted whose parents or guardians are not prepared to allow him or her to remain for at least three months, and only in exceptional circumstances will a child be kept for more than twelve months." This proviso is necessary because it is undesirable that any child should be given a limited chance of permanent improvement by merely a few weeks' stay; three months is the least period in which permanent benefit can be hoped for, considering the fact that a short period must elapse before the new comer falls into the routine of the School and becomes familiar with its methods. A stay for a period longer than one year is undesirable since it tends to make the Home into an asylum, a contingency that should be strictly guarded against. Children who derive no visible benefit from a stay of six months are best treated in some other fashion; they ought to have hospital treatment or to go to a sanatorium where more active medical treatment can be given.

### Method of Admission.

The Trustees make it a condition of admission that no child should be admitted or allowed to remain in the Home who suffers from or show signs of active and infectious or chronic consumption of the lungs or throat or other parts of the body, or from any other infectious or contagious disease, or who is mentally deficient or morally degenerate, or subject to or suffers from fits or epilepsy, or anything which makes it undesirable that he or she should mingle or associate with other children. These provisos are self-evident and do not impose unnecessary restrictions on the selection of candidates for admission when it is remembered that the School is intended for feeble and not for actively sick children. The Trustees further state that they prefer to obtain their children through a proper educational, Poor-Law, or health authority, who must make a grant proportionate to the time for which such a child may remain an inmate of the Home, and at a rate to be mutually agreed upon. A medical certificate must accompany each form of application. Cases which it is decided to admit will be registered, and when they can be received an order of admission will be sent. At the same time applications for the admission of children from private sources will be considered on their merits on terms to be mutually arranged. The institution is, of course, strictly undenominational.

This short summary of the objects and aims of the new institution will give the reader some idea of its scope and intention. That there is need for such an institution, great need, there can be no doubt whatever, and it will be interesting to follow the work of the Home and see what its results are. Under proper management and with careful selection there is no reason why these results should not be admirable, and prove an incentive to other philanthropic bodies, public as well as private, to emulate the wise precedent of the Ogilvie Trustees and establish similar homes in other parts of the country. From a structural point of view the building is admirably fitted for its purpose, great care having been taken to make the plans complete, and to build the Home under conditions that compare excellently with those that obtain in German and American establishments of a somewhat similar nature. The general manager of the Ogilvie Charities is Mr. Charles Smith, of 26 Lincoln's Inn Fields, London, from whom application forms for admission into the Home and all further particulars may be obtained.

